# DNA Directed Pro-Dopamine Regulation Coupling Subluxation Repair, H-Wave^®^ and Other Neurobiologically Based Modalities to Address Complexities of Chronic Pain in a Female Diagnosed with Reward Deficiency Syndrome (RDS): Emergence of Induction of “Dopamine Homeostasis” in the Face of the Opioid Crisis

**DOI:** 10.3390/jpm12091416

**Published:** 2022-08-30

**Authors:** Anish Bajaj, Kenneth Blum, Abdalla Bowirrat, Ashim Gupta, David Baron, David Fugel, Ayo Nicholson, Taylor Fitch, B. William Downs, Debasis Bagchi, Catherine A. Dennen, Rajendra D. Badgaiyan

**Affiliations:** 1 Bajaj Chiropractic Clinic, New York, NY 10010, USA; 2 Division of Addiction Research & Education, Center for Psychiatry, Medicine, and Primary Care, (Office of the Provost), Western University Health Sciences, Pomona, CA 91766, USA; 3 The Kenneth Blum Institute on Behavior & Neurogenetics, Austin, TX 78701, USA; 4 Department of Molecular Biology, Adelson School of Medicine, Ariel University, Ariel 40700, Israel; 5 Future Biologics, Lawrenceville, GA 30043, USA; 6 Department of Psychiatry, South Texas Veteran Health Care System, Audie L. Murphy Memorial VA Hospital, Long School of Medicine, University of Texas Medical Center, San Antonio, TX 78229, USA; 7 Department of Pharmaceutical Sciences, Southern University College of Pharmacy, Houston, TX 77004, USA

**Keywords:** H-Wave, Genetic Addiction Risk Severity (GARS), Reward Deficiency Syndrome (RDS), cognitive behavioral therapy, dopamine, KB220PAM, subluxation, resting state network, proprioception, photobiomodulation

## Abstract

Addiction is a complex multifactorial condition. Established genetic factors can provide clear guidance in assessing the risk of addiction to substances and behaviors. Chronic stress can accumulate, forming difficult to recognize addiction patterns from both genetic and epigenetic (environmental) factors. Furthermore, psychological/physical/chemical stressors are typically categorized linearly, delaying identification and treatment. The patient in this case report is a Caucasian female, aged 36, who presented with chronic pain and partial disability following a surgically repaired trimalleolar fracture. The patient had a history of unresolved attention deficit disorder and an MRI scan of her brain revealed atrophy and functional asymmetry. In 2018, the patient entered the Bajaj Chiropractic Clinic, where initial treatment focused on re-establishing integrity of the spine and lower extremity biomechanics and graduated into cognitive behavior stabilization assisted by DNA pro-dopamine regulation guided by Genetic Addiction Risk Severity testing. During treatment (2018–2021), progress achieved included: improved cognitive clarity, focus, sleep, anxiety, and emotional stability in addition to pain reduction (75%); elimination of powerful analgesics; and reduced intake of previously unaddressed alcoholism. To help reduce hedonic addictive behaviors and pain, coupling of H-Wave with corrective chiropractic care seems prudent. We emphasize the importance of genetic assessment along with attempts at inducing required dopaminergic homeostasis via precision KB220PAM. It is hypothesized that from preventive care models, a new standard is emerging including self-awareness and accountability for reward deficiency as a function of hypodopaminergia. This case study documents the progression of a patient dealing with the complexities of an injury, pain management, cognitive impairment, anxiety, depression, and the application of universal health principles towards correction versus palliative care.

## 1. Introduction

### 1.1. The Opioid Crisis

Noncancerous pain treatment presents a challenge for primary care medicine. The United States of America (USA) has experienced iatrogenically induced opiate fatalities. In 2021, north of 100,000 Americans died from unintentional opioid-induced overdoses [1,2]. The increased usage of prescription opioid analgesics has been a major contributor to the rise in drug overdose mortality rates. Although opioid analgesics were initially responsible for more overdose deaths than heroin and cocaine combined [3,4], the current availability of inexpensive street opiates has increased the dependence on heroin [5,6,7]. A National Institute of Health (NIH) survey estimated that by 2014, 25.3 million adults suffered with daily pain for the previous three months. In 2016–2017, several thousands of people died from opioid/opiate overdose, specifically from the synthetic opioid fentanyl. In 2016, to mitigate this rising threat to the public, new guidelines for prescribing opioids to patients suffering with chronic pain were issued by the Center for Disease Control (CDC). In 2017, morphine milligram equivalents fell by 29%, but more than hundreds of thousands of individuals still died from narcotic overdoses, resulting in a decrease in the national life expectancy.

According to the National Institute on Drug Abuse (NIDA), about 116 million Americans currently suffer from chronic pain. Individuals with chronic pain are also more likely to suffer from a variety of mental and physical health conditions, including an array of addictive behaviors. Although significant monetary fines into the billions have been levied on pharmaceutical companies, the use of opioids and other drugs of abuse continue to contribute to this ongoing crisis. 

### 1.2. Pain Demographics

Every 14 minutes, 150 million individuals are negatively affected by and suffer from pain conditions. Yearly, around 300 million narcotic prescriptions are filled, costing hundreds of billions of dollars. Some of these patients die from prescription overdose. It is well known that the consumption of potent narcotics to alleviate pain, can result in higher tolerances and severe withdrawal symptoms within a relatively short period of time [8]. A website explaining the impact of chronic pain in the USA can be found at https://www.cdc.gov/mmwr/volumes/67/wr/mm6736a2.htm (accessed on 3 January 2021).

“Reward Deficiency Syndrome” (RDS) [9] is a genetically based form of hypodopaminergia that affects about one-third of the United States population [10]. Some individuals can tolerate potent narcotics and do not crave or want opioids after treatment for pain or even withdrawal. Others, however, become enthralled with addictive-like behaviors once their treatment course has finished and their pain is alleviated, which is often the result of genetic and epigenetic insults [11]. Our group recently published a study utilizing the Genetic Addiction Risk Severity (GARS) test that revealed a high drug and alcohol risk in probands who attend and are chronically prescribed opioids at multi-pain clinics. The continuous requirement for powerful narcotics may be influenced by genetic predisposition, especially in chronic pain conditions [12]. It has been shown that chronic pain disrupts functional brain connectivity in areas unrelated to pain which are known to be active at rest, namely, the default mode network, further underscoring the broad impact of pain on cognition and behavior.

### 1.3. Substance and Non-Substance Use and Misuse Is a Global Societal Pandemic

European regions were found to have the highest prevalence rates of heavy episodic alcohol use and daily tobacco use. The age-standardized prevalence of alcohol dependence was 843.2 per 100,000 people; for cannabis, opioids, amphetamines and cocaine dependence it was 259.3, 220.4, 86.0 and 52.5 per 100,000 people, respectively. In North America, high-income areas had some of the highest rates of cannabis, cocaine and opioid dependence. Attributable disability-adjusted life-years (DALYs) were found to be the highest for tobacco smoking (170.9 million DALYs), followed by alcohol use (85.0 million) and illicit drug use (27.8 million). Substance-attributable mortality rates were the highest for tobacco smoking (110.7 deaths per 100,000 people), followed by alcohol and illicit drug use (33.0 and 6.9 deaths per 100,000 people, respectively). Attributable age-standardized mortality rates and DALYs for alcohol and illicit drug use were found to be the highest in eastern Europe; attributable age-standardized tobacco mortality rates and DALYs were the highest in Oceania [13]. In terms of gaming disorder, the worldwide prevalence was 3.05%. Gaming disorder rates were also ~2.5:1 in favor of males compared to females [14].

Unfortunately, amidst the current COVID-19 pandemic and associated variants, seeking addictive behaviors, both substance and non-substance continue to increase with related opioid overdose above pre-COVID levels [15]. Pain management experts believe that a high number of opiate overdoses are the result of patients trying to manage their unrelenting pain and are in fact not intentional [16].

It is noteworthy that the most comprehensive study of the newly established overlapping neurogenetic basis for chemical and behavioral addictions alike, by Kotyuk et al. [17], involved 3003 young adults. The study revealed strong associations between smoking and alcoholism with excessive internet use, gambling, and cannabis use. The Kotyuk et al. [17] data support the concept of Reward Deficiency Syndrome (RDS), a featured disorder in the SAGE Encyclopedia of Clinical and Abnormal Psychology [18,19]. The concept of RDS, first coined by Blum in 1995, has been identified as cytoarchival evidence for the basis of all addictive behaviors [20] and translates to the component model of addictions that proposes a common phenomenological and etiological background of various addictive behaviors [20]. There are also independent data to support this concept [21,22].

### 1.4. Why Electrotherapy for Pain

In the United States, abuse related to iatrogenic prescription drugs is the fastest escalating drug issue. Two major populaces at-risk in the USA related to prescription drug overdose are nine-million individuals reporting long-term medical opioid usage and five-million individuals reporting non-medical usage. Of the individuals who are prescribed high daily doses, 20% are receiving care under many clinicians. These individuals account for about 80% of overdose reports and are more susceptible to share the prescribed substances with others who use them without any prescription [23]. 

The central pathways that stem from the dorsal horn of the spinal cord to the medulla along with several genes and their biomarkers inhabiting the mesolimbic reward center of the brain play a role in controlling pain tolerance and sensitivity [24,25,26].

Recognizing the reward genes along with their polymorphisms might offer unique biomarkers to non-narcotic pharmacogenomic modalities to alleviate pain. The GARS test [27] has the potential to identify patients at the earlier stages of treatment who are prone to addiction; for instance, reward gene alleles such as DRD2 A1 and the G allele of the Mu Opioid Receptor are correlated with chances of addiction to narcotics. Such individuals will need non-addictive treatment modalities to mitigate pain. The electrotherapeutic device, H-Wave^®^ (Electronic Waveform Lab Inc., Huntington Beach, CA, USA) is an example of such alternative treatment modality [28].

### 1.5. The Characteristics of H-Wave^®^ Electrotherapy

The mechanisms of action of the H-Wave device stimulation (HWDS) assessed physiologically in a pre-clinical model included decreased edema attributed to the stimulation of smooth muscle fibers within the lymphatic vessels [29]. Furthermore, HWDS stimulates nitric oxide (NO)-dependent microcirculation increase as well as angiogenesis resulting in tissue healing. 

The HWDS characteristics include:

· · Low frequency (1–2 Hz) stimulation-induced contraction of smooth and skeletal muscle (red, slow-twitch) fibers leads to tissue loading while retaining the characteristics of low muscle force tension or non-fatiguing by avoiding tetanizing contractions;
· · NO-dependent arteriolar vasodilation (revealed by rat studies);
· · Bromouridine staining showed enhanced angiogenesis in repetitive stimulation in rats;
· · Fluid shifts and reduced edema and protein clearance caused by direct stimulation of smooth muscle fibers in the lymphatic vessels

Nonpharmacological alternatives are required to mitigate pain amid the current opioid crisis. It is well documented that opioid use can lead to respiratory depression [30]. Reduced respiratory drive and resulting low oxygen levels hinder systemic cellular function let alone the healing of injured areas. Given our functional dependence on oxygen for survival, sustainable modalities such as H-Wave which target supporting circulation could play a broader role in both early injury intervention and recovery from chronic pain states. Over 18 studies including original articles, review articles, and abstracts are published in peer-reviewed journals illustrating the positive effects of H-Wave including mechanism of action and pain relief [28,29,31,32,33,34]. Amidst our terrible opioid crisis, with several individuals losing their lives daily, alternatives to strong pain medications need to be adopted by the entire analgesia society. 

Over the course of the past two decades, investigators have been increasingly keen in managing pain and restoring function by the use of electrical stimulation. One of the focal points of interest is the use of the H-Wave^®^ device [28,29]. The objective of the HWDS is to reduce chronic pain and inflammation. This can be achieved by [28]:

- Direct stimulation of the smooth muscles of lymphatic vessels and small-diameter skeletal muscle fibers by low-frequency (1–2 Hz) stimulation resulting in interstitial fluid shifts. Long rhythmical contractions of these particular muscles caused by HWDS lead to decline in accretion of inflammation-associated proteins, an essential part of pain and associated disability in chronic injury or trauma patients.
- HWDS at high frequency (60 Hz) affects the function of sodium pumps in nerves leading to analgesic and/or anesthetic effects.
- NO-dependent stimulation of skeletal muscles induced by HWDS results in significant microcirculation increase, as evident from preclinical studies.
- Angiogenesis causes a profound and rapid increase in blood flow, which is seen in rat hind limbs post-repetitive HWDS.

Based on the data, it can be reasonably assumed that repetitive HWDS can reduce inflammation and aid in quicker healing and better recovery, owing to reduced protein accumulation in conditions such as post-operative rotator cuff reconstruction. 

A meta-analysis by Blum et al., systematically reviewed the HWDS safety and efficacy for treating chronic inflammation of neuropathic and soft tissue pain. It included five studies linked to pain alleviation, decrease in utilization of pain medication, and improved function. Data were examined using the random-effects model, including correction to assess variability, study size, and bias in effect size [29]. This study utilized data from a total of 6535 patients [29,31,32,33,34]. Although there is a moderate-to-strong effect of the HWDS in offering pain relief, reduction in pain medication usage, and improved function as reported in this study, additional studies are warranted. The best result was noted for improved function, indicating that the HWDS can lead to a speedier return to work and other associated daily activities [35]. The presence of high muscle spindle densities in the spine, hands and feet may underlie the importance of proper whole-body mechanoreception including balance, breathing and other essential posture-related functions. Combining chiropractic adjustments and H-Wave to both the spine and extremities may simulate a more complete activation and recovery cycle in subluxated body regions presenting with fixation, misalignment or other movement deficiencies. Integrating care modalities that compromise the healing process while guiding the focus of care on complete recovery of functional capacity over analgesia alone.

### 1.6. Why Genetic Addiction Risk Severity (GARS) Test?

On the one hand, pain specialists face the risk of their patients being dishonest about their true pain level or sensitivity because they are stuck within the “addictive process”, which may be linked to genetic biomarkers that are associated with the reward circuitry. On the other hand, patients need powerful narcotics in order to circumvent disruptive pain-related symptoms. The challenge is in establishing a way to discriminate between these two types of patients at the start of their treatment and genetic testing could provide the answer. Though this appears to be a simple concept, we must contemplate that our DNA may predispose individuals to addictive-like behaviors in addition to the environment, specifically, epigenetic processes that influence the expression of genes [36]. According to a PubMed search performed on 3 January 2022, there are at least 49 reviews and original studies on GARS. Unfortunately, the majority of these articles are from our group; however, we encourage others to independently substantiate these preliminary findings [9].

**Figure 1:**
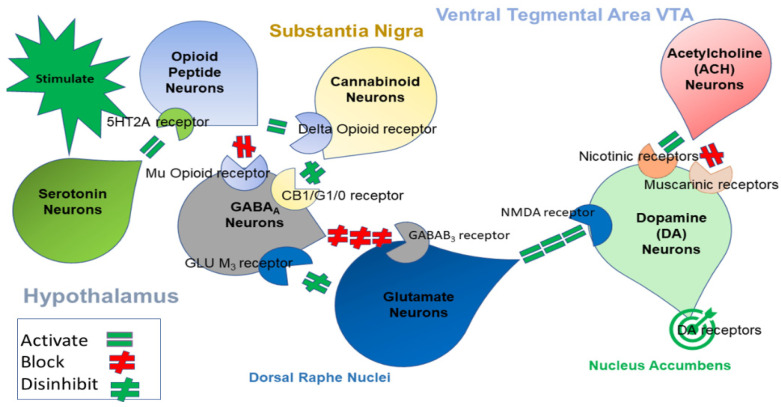
Illustration showing the interaction of at least seven major neurotransmitter pathways in the complex of the Brain Reward Cascade (BRC). In the hypothalamus, environmental stimulation springs the release of serotonin, which in succession via, for example, 5HT-2a receptors activate (equal green sign) the ensuing release of opioid peptides from opioid peptide neurons, also occurring in the hypothalamus. Afterwards, the opioid peptides have, potentially via two different opioid receptors, two distinct effects: one that inhibits (red hash sign) through the mu-opioid receptor (possibly via enkephalin) and projects to the Substantia Nigra to GABAA neurons; or the other, which stimulates (equal green sign) cannabinoid neurons (the Anandamide and 2-archydonoglcerol, for example) via Beta-Endorphin-linked delta receptors, which in turn inhibit GABAA neurons at the Substantia Nigra. Additionally, when activated, cannabinoids, largely 2-archydonoglcerol, may indirectly disinhibit (red hash sign) GABAA neurons through activation of G1/0 coupled to CB1 receptors in the Substantia Nigra. In the Dorsal Raphe Nuclei, glutamate neurons can indirectly disinhibit GABAA neurons in the Substantia Nigra through activation of GLU M3 receptors (red hash sign). GABAA neurons, when stimulated, will, in turn, intensely (red hash signs) inhibit VTA glutaminergic drive via GABAB 3 neurons. It is also feasible that stimulation of ACH neurons at the Nucleus Accumbens ACH will stimulate muscarinic (red hash) or Nicotinic receptors (green hash). Lastly, Glutamate neurons in the VTA will project to dopamine neurons by way of NMDA receptors (equal green sign) to preferentially release dopamine at the Nucleus Accumbens, depicted as a bullseye which indicates a euphoria or “wanting” response. The outcome is that when dopamine release is low (endorphin deficiency), unhappiness is experienced, while general (healthy) happiness is dependent on the dopamine homeostatic tonic set point. (With permission from Blum et al.) [20].

Ultimately, in today’s society, countless people are dying as a result of legal and illegal narcotics, and state laws, government organizations and “big pharma” make it extremely difficult for chronic pain patients to receive the appropriate treatment required [37]. Knowing a patient’s GARS score is likely to improve care by providing a more in-depth view of a patient’s addiction risk and removing preconceptions related to addiction. Thus, once more randomized control (RCT) studies are completed, the hypothesis is that pain reduction utilizing H-Wave therapy and Chiropractic care, without the use of addictive analgesics, will be a laudable goal for patients with addiction vulnerability/liability, as determined by GARS testing. Along these lines, our laboratory in conjunction with scientists from Geneus Health LLC (San Antonio) developed the GARS test to help identify RDS in terms of DNA risk biomarkers of genes linked to the brain reward circuitry, as depicted in Figure 1. In 1989, Blum and Kozlowski [38] published their initial concept of the Brain Reward Cascade. In previously published works from our laboratory [20], we proposed a Brain Reward Cascade (BRC) schematic at mesolimbic sites, as described in Figure 1. 

The various genes and associated polymorphisms linked to the ten genes and eleven alleles measured in the GARS test yield knowledge related to a hypodopaminergia in the brain reward circuitry. The phenotype is best expressed by the construct referred to as Reward Deficiency Syndrome (RDS). To date (6 February 2022, there are 1436 articles listed in PubMed using the term “Reward Deficiency” and 220 using the term “Reward Deficiency Syndrome (RDS)”, whereby 47% are independent of Blum’s laboratory.

### 1.7. Why Pro-Dopamine Regulation (KB220)

Processing of endogenous endorphins or response to exogenous dopamine stimulants may be interrupted/impaired in people with addictive, impulsive, compulsive, and certain personality disorders. RDS is a polygenic trait with associations that indicate cross-talk between various neurological systems, including the well-known reward pathway, motivational systems, and neuroendocrine systems. Animal models utilized to research substance use disorder (SUD), depression, early life stress, immune dysregulation, PTSD, ADHD, compulsive eating disorders, and compulsive gambling were previously discussed [39]. 

The complex of mental health co-morbidities and their related addictive human behaviors may be better explained by universal metabolic imbalances offered by the reward deficiency model [39]. These disorders recruit underlying reward deficiency mechanisms in multiple areas of the brain. The basic phenotype recognized as RDS has an extensive and remarkable array of correlated/overlapping disruptive behaviors with a common cause of hypodopaminergia. The cognitive implication of such recruitment is demonstrated in EEG studies of obsessive–compulsive subjects presenting with deficits in memory and selective attention [40]. Further, patients with chronic low back pain have shown decreased default mode network disruptions on fMRI, while brain morphometrics revealed a correlation between chronic back pain and cortical atrophy [9,12].

It is worth noting that the pursuit of pleasure and fulfillment, as opposed to essential survival necessities and chronic indulgence, is timeless and has been debated and discussed for millennia. Epicurus of Samos (341–270 BC), a Greek philosopher, advocated for a hedonistic calculus to achieve balance and happiness, which included accounting for both mental and physical experiences of pleasure and pain [41]. It is now known that feelings of fulfillment, well-being, and accomplishment after completing a task are mediated by natural neurotransmitters which are released in the brain’s reward centers, forming a functional network primarily comprising the midbrain, cerebral cortex, and limbic system, referred to as mesocorticolimbic [42]. Berridge and Kringelbach investigate theories of pleasure such as love, desire, disgust, euphoria, and anhedonia, which are also hypothesized to share a common currency by brain reward-processing systems [42,43]. 

Many mental health illnesses represent characteristics or situations where ‘satisfaction’ or ‘elation’ is difficult to achieve due to an imbalance of neurotransmitters (i.e., 5-hydroxytryptamine (serotonin), norepinephrine, dopamine, glutamate, GABA) and neuropeptides (endorphins), which increases the chemical requirement needed to compute pleasure and reward within the mesolimbic system [44,45]. Addictions to drugs, food, gambling, sex, etc., all have the same “brain reward” imbalances, which are caused by insufficient dopamine release or ineffectual dopamine processing mechanisms, resulting in a hypodopaminergic state [46,47,48]. Epicurus was most likely referring to achieving a balance of the biologically defined ‘brain reward’ from both a physiological and philosophical perspective long before these models were supported with empirical neuroscience evidence. There are numerous psychiatric disorders that are linked with impaired dopamine homeostasis, and the complicated mechanistic bases for these disorders are intensively researched [49].

KB220 has been investigated in several clinical trials and cases of RDS patients [50,51,52,53], as well as in rats to understand its impact in brain dopamine circuits [54]. A randomized placebo-controlled crossover study in humans indicated that KB220Z had a putative anti-craving/anti-relapse effect. However, due to the small sample size, final interpretation of the results was limited, necessitating further human and rodent studies of KB220Z [51,52,53]. In an additional study [55], Gondre-Lewis’s team [55] used P adult male and female rats to test not only the effects of KB220Z on binge drinking, but also the most effective route of administration: oral (P.O.), subcutaneous (S.Q.) and intraperitoneal (I.P.). Data acquired from their investigation support the hypothesis that KB220Z reduces alcohol consumption and associated behavioral deficiencies, such as psychological sequelae of open field activity and P rats’ exploration of the EZM open area. It is widely recognized that hypodopaminergic reward circuitry causes imbalances in dopamine homeostasis. This imbalance results in drug-seeking behavior and other associated behavioral deficiencies. Furthermore, they suggested that KB220Z acts via a potent pro-dopamine regulator, assisting in the maintenance of optimal dopamine levels within the reward circuit, resulting in decreased ethanol cravings and consumption. This nutraceutical intervention, which utilizes a nutrigenomic approach, may be useful alone or in combination with conventional treatment regimens, and could help SUD patients maintain abstinence for longer periods of time. The importance of dopaminergic genetics and RDS must be recognized [56,57,58,59]. There are at least 50 peer-reviewed articles examining the effects of KB220 to date [60].

### 1.8. Precision Addiction Management: The Future Is Now

Millions of people throughout the world are unable to overcome their frustrating and often fatal love affair with getting high; for some, “high” may simply mean experiencing feelings of happiness. The neuroscience community undertakes and funds excellent research utilizing advanced neuroimaging and molecular-genetic-applied technology to enhance understanding of the complex functions of brain reward circuitry, which plays an important role in addiction symptomatology. While it is commonly acknowledged that dopamine is a key neurotransmitter involved in substance and behavioral addictions, there is still controversy regarding the appropriate utilization of dopamine in therapeutic settings to prevent and treat various kinds of addictive disorders. A biphasic approach with short-term blockage followed by long-term dopaminergic upregulation, could be beneficial. The objective of treatment would be to increase brain reward functional connectivity volume, as well as target reward deficiency and the stress-like anti-reward symptomatology of addiction. These phenotypes could be characterized utilizing the GARS test. Dopamine homeostasis can thus be attained with “Precision Addiction Management” (PAM), which involves customizing neuronutrient supplementation (KB220), based on the results of the GARS test, in conjunction with behavioral intervention (e.g., Cognitive Behavioral Therapy). For pharmacokinetic challenges, GARS results were evaluated neurogenetically and neuropsychologically, and used as a roadmap for developing a distinctively informed genomic nutraceutical non-pharmaceutical intervention, tailored specifically to mitigate increased dopamine metabolism and increased serotonin re-uptake. The daily pro-dopamine treatment was gradually reduced and subsequently terminated at 80 days of abstinence and was only occasionally utilized as an “emergency assist” for short-term relief when the severity of RDS symptoms threatened reinstatement. Personalized for phenotype and guided by DNA, the Pro-Dopamine regulator (KB220Z), also known as Neuroadaptagen Amino-Acid Therapy (NAAT), was developed to restore brain health. The utilization of this customization is based on a priority algorithm and also covered by patents in the USA and patents pending globally. 

## 2. Case Presentation

Addiction is a complex condition that affects the central nervous system and many related areas. There are established genetic risk factors that which when identified can give clear guidance in assessing risk to exposure of substances and behaviors. For every clear indicator of risk there may be dozens of combinations of risk indicators which are more difficult to recognize, including both genetic and epigenetic (environmental and behavioral-stress-induced), giving rise to hidden addictions that are even more difficult to unravel. Furthermore, stressors coming from psychological, physical, and chemical sources have been categorized linearly, which has slowed identification and risk of addiction from multiple sources—another roadblock to efficient and effective care. Although this is a case study, the patient did sign a PATH Foundation IRB approved (2017) consent form indicating an exempt status for the procedures to follow. 

### 2.1. Treatment Methods

#### Clinical History

**Figure 2:**
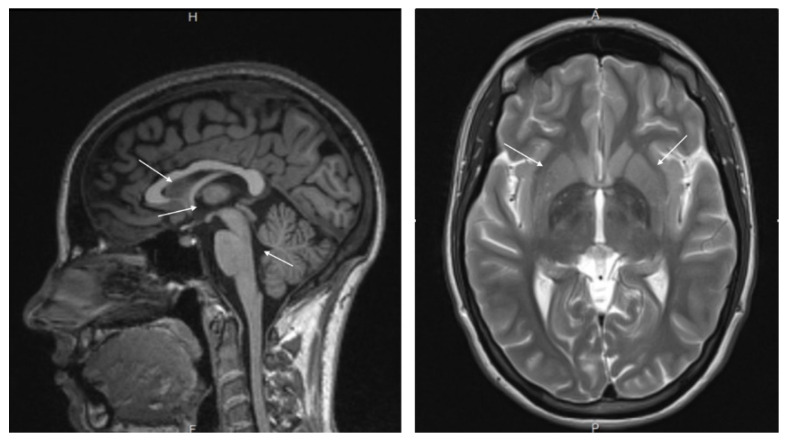
MRI showing atrophy, and functional asymmetry.

The patient is a Caucasian female, aged 36, presenting with a Trimalleolar Fracture and a dislocated right ankle that occurred in 2/24/2015 from a fall. The patient subsequently underwent surgery in May 2015 to repair the fracture, followed by limited physical therapy, and prescription pain medications to help eliminate the pain associated with her partial disability. During recovery from injury, the patient’s medicine management expanded to address the following pharmacological agents: sleep, anxiety and depression. It is noteworthy that the patient had previous established history of ADD with medical management, which was unresolved. An MRI scan of her brain revealed atrophy and functional asymmetry (Figure 2). The patient’s pharmaceutical care escalated to include Methylphenidate since she was 19-years-old with a sequalae of unwanted side effects. 

### 2.2. GARS Test and Imaging Diagnostics 

In terms of the rationale, the Genetic Addiction Risk Score (GARS) was utilized to explore a clearer neurogenetic basis for the patient’s complex clinical presentation and support efforts to reduce harm in substance and behavior choices. The breadth of biological specific mechanisms explained in the GARS profile offer insights into both problematic reward patterns as well as validation of more sustainable and natural reward processes sought in this case and the many that it represents. The scientific evidence for GARS involves at least 58 PubMed listed articles including original research, one example is by Blum et al. [27]. The selection of genes in the GARS test can be found by referring to work of Blum et al., published over almost a decade ago [40]. Other studies provide important evidence linking many RDS behaviors and associated psychological constructs including reward gene polymorphisms as measured in GARS [61,62,63].

### 2.3. Imaging Methods

#### 2.3.1. MRI Brain

MRI examination of the brain was performed on a Siemens Trio 3.0 Tesla scanner utilizing sagittal 3 mm 3D T1, axial 4 mm gapless fat-suppressed T2/FLAIR, axial 4 mm gapless T2, coronal 3 mm gapless fat-suppressed T2, axial 2 mm gapless 3D susceptibility weighted imaging (SWI) for hemosiderin detection, and two-dimensional diffusion-weighted imaging.

#### 2.3.2. DTI Brain

Diffusion-tensor imaging (DTI) was performed on a Siemens Trio 3.0 Tesla scanner utilizing 30 directions with 5 mm sections, 1.5 mm gap, 230 mm field of view, TE/TR 106/3000 ms, two averages, AP phase, 128 × 128 matrix, 1.8 × 1.8 × 5.0 mm voxel size, GRAPPA acceleration factor 2, 38 reference lines, b value 2000, bandwidth 1396 Hz/Px and EPI factor of 128. After informed consent was obtained, control subjects were scanned at the same facility on the same magnet, using the same technique. All controls subjects provided a negative history for psychiatric or neurologic disorder. No information regarding smoking history or non-neurologic problems was obtained. After removal of some control subject data due to technical factors, the remaining 109 control subject DTI examinations were analyzed. For DTI scans, eight ROI regions were drawn in house (see table below) and fractional anisotropy (FA) was analyzed. The mean control FA, standard deviation, and lower limit of normal patient FA were calculated for Z scores of −1.282 and −1.645 corresponding to *p* < 0.10 and *p* < 0.05, respectively.

#### 2.3.3. NeuroQuant

Sagittal 3D T1-weighted MPGR was performed utilizing ADNI protocol with 1.2 mm isotropic voxels on a Siemens Trio 3.0 Tesla scanner. DICOM data were then analyzed utilizing FDA-approved NeuroQuant (version 2.0) from Cortechs Labs (San Diego, CA, USA) utilizing a dynamic atlas based upon >2000 age- and gender-matched controls. Cortical thickness in 50 regions (25 per hemisphere), ventricular, and hippocampal volumes are measured; percentile rank and Z-score are reported.

#### 2.3.4. MR Angiogram (MRA)

A 3D time-of-flight MR angiogram of the brain was performed on a Siemens Trio 3.0 Tesla scanner utilizing 0.6 mm isotropic voxels followed by 3D multiplayer reformation.

### 2.4. Therapeutics 

In this case study, we utilized a multi-therapeutic protocol to not only overcome unwanted chronic pain but a number of related RDS behaviors, including addiction. These included: 

(1) *Subluxation*—The patient received a bio-mechanical assessment that included examination of spine-related functions, a posture screening and a digital weight-bearing foot exam. The examination findings, rather than symptoms alone, are the determining factors in the starting point for the progression of care. Adjustments of subluxation were performed primarily on the spine, as well as related areas including the cranium and upper and lower extremities with a focus on restoring and maintaining balance and respiration function. One interesting article related to the hypothesis of subluxation and pain was authored by Brantingham [64] along with 7712 other articles listed in PUBMED (accessed on 7 January 2022); 

(2) *H-Wave^®^ device stimulation*—This multi-functional electrical stimulation device was used with the intention of facilitating both functional improvement and pain management without harmful side effects. One protocol targeted non-invasive drug-free recovery from chronic and post-operative pain. H-Wave was also utilized to accelerate restoration of function through increased blood circulation and lymphatic drainage while improving ranges of motion and balancing muscle tone [28]; 

(3) *Custom functional orthotics*—Ideally, flexible, strong arches of the feet are developed in childhood and maintained throughout life. Some people never develop proper arches, while others may present with sagging of the foot’s arch, or pes planus, as a result of repetitive stresses from daily activities which gradually lengthen the connective tissues. Normal arches not only provide support for the bones and joints of the feet but also provide stability for the spine and pelvis. Decreases in elasticity of the arch are difficult to restore resulting in the requirement of Custom Functional Orthotics. Three-dimensional scans of the patient revealed bilateral asymmetrical pronation and provided the basis for individually designed custom orthotics [65]; 

(4) *Amino-acid-based -Enkephalinase inhibition supplementation*—KB220PAM was considered for the pro-dopamine regulation it offers as demonstrated through extensive research in human clinical settings. Through specialized neuro-imaging analysis, the mechanism of action (MOA) was recently illuminated [66]. QEEG studies demonstrated smoothing out of dis-regulated PFC cingulate gyrus one hour after administration of KB220Z to alcoholic patients, psycho-stimulant-addicted individuals and heroin-addicted individuals [66]. The neuro-nutrient’s regulation of brain electro-activity through increased alpha and low beta waves offer elusive stability compared to placebo (triple blinded). This region of the brain has been associated with reward relapse (e.g., sugar indulgence, smoking, drug use, etc.). Additional qEEG studies linked to issues related to opioids, alcohol and other psychoactive drugs have also demonstrated significant benefits clinically in humans [67,68,69,70]; 

(5) *Cognitive Behavioral* Therapy—Cognitive behavioral therapy (CBT) is a form of intentional psychological treatment that has been shown to be effective for a wide range of issues including, but not limited to, anxiety disorders, depression, alcohol and drug use. The evidence for efficacy of CBT has been thoroughly reviewed and was considered in this case for the significant improvement demonstrated in numerous studies on functioning and on quality of life. CBT has been shown to be as, if not more, effective than other less proactive forms of psychological therapy or psychiatric medications [71]; 

(6) *Photobiomodulation*—Photobiomodulation (PBM) treatment, which involves light therapy utilizing non-ionizing light sources in the visible and infrared spectrum, was sought for its potential to alleviate pain and inflammation. This non-thermal application engages endogenous chromophores that elicit photochemical and photo-physical events that may lead to pain relief as well as promotion of tissue regeneration and wound healing [72].

## 3. Diagnostic Results 

### 3.1. MRI and MRA Results

Findings included significant FA reduction within the centrum semiovale bilaterally, significant cortical atrophy on the left side, and mild increase in ventricular size. Combination of findings is suggestive of traumatic brain injury, psychiatric disorder or toxic encephalopathy in view of the younger age of this patient. Findings from the brain MRA revealed no abnormalities. Clinical correlation is needed (see Figure 2). 

In 2018, the patient entered the Bajaj Chiropractic Clinic (New York, NY, USA). The patient’s treatment included a multi-array of therapeutic modalities which included: Chiropractic Adjustments, H-Wave Therapy, Cognitive Behavioral Therapy, Photo-biomodulation, Custom Functional Orthotics and GARS test to help provide precision-guided DNA Pro-dopamine regulation (KB220PAM) to potentially induce “dopamine homeostasis”. It is not our intent to perform imaging after the proposed treatment success. 

### 3.2. GARS Results

The patient undertook the DNA swab GARS test and scored an eleven (11) which displays a very high risk for RDS behaviors. Specifically, the test results are as follows:

COMT-rs4680 with risk allele G showing A/G with a score of 1. Additional copies of the Val variant results in increased reaction rates of catechol-O-methyltransferase, which metabolizes dopamine in the synapse, reducing reward functions related to its presence.

DRD2/ANNKKI-rs1800497 with risk allele A1 showing A1/A2 with a score of 1. This genetic variant is associated with reduced D2 dopamine transduction resulting in a decreased sense of well-being normally experienced with common behaviors such as food consumption, reproduction and work.

DRD4-rs1800955 with risk allele C showing C/C with a score of 2. 

DRD4-rs7610108847 with risk allele ≥ 7 repeats showing 3R/7R with a score of 1. The presence of the ‘long’ risk form of the variant reduces activated D2, which is similar in function to D2 receptors which inhibit adenylate-cyclase-mediated conversion of ATP to cyclic AMP.

OPRM1-rs1799971 with risk allele G showing G/G with a score of 2. The G allele engenders those with the variant with a reduced response to opioids, due to inhibition of mu opioid receptors, resulting in inadequate pain relief and higher analgesia thresholds.

HTT-LINKED-rs4795541 with risk allele S, LG showing S/LA with a score of 1. Excessive serotonin recycling rates, secondary to long allele LA causes reduced serotonin signaling and related predisposition to depressive responses to stress.

MAOA-rs768062321 with risk allele 3.5R,4R showing 3R/4R with a score of 1. The MAOA gene 4A variation is associated with excessive enzyme break down of dopamine resulting in reduced DA signaling and increased RDS behaviors.

GABRB3 -rs764926719 with risk allele 181 showing 181/181 with a score of 2. Allele 181 increases net GABA-A activity, hence greater inhibition of neuronal activity and low-dopamine function.

Based on a number of studies, it has been established that for carriers of ≤ any 4 alleles there is a risk for drug abuse and carriers of ≤ any 7 alleles, there is a risk for alcohol abuse [12,27,73]. The patient reported difficulty with alcohol abuse as well as with controlling opioid anti-pain medications [25]. 

## 4. Clinical Outcome

During the course of treatment from 2018 to 2021, the patient’s care progressed through several phases keyed by unique clinical integrations. Phase one focused on correction of ongoing maladaptive changes secondary to the ankle injury by addressing spinal subluxation and extremity misalignments through chiropractic care and the need for increased blood flow with H-Wave therapy. In terms of pain reduction, the pain-score of 8 reduced to a pain-score of 2 (75% reduction); elimination of powerful analgesics. As of March 2019, painkillers were reduced in favor of nutrition and natural supplementation; however, chronic use of other medicines persisted. To this end, the patient started a healthcare coaching program using the Emotional Freedom Technique (EFT) with a dedicated coach to help organize her thoughts. The EFT coach’s view of patient’s initial presentation was that the patient’s emotional distress was focused on her inability to stay focused, think and perform clearly and consistently, as well as coping with the trauma of her ADD diagnosis. In terms of her ADD issue, the patient self-reported that she was having the following challenges due to pain medications prescribed to her after the injury and the Adderall prescription prescribed to her since age 19 to manage her ADD: diminished attention, inconsistent performance, irritability and mood swings with risky behavior, extended periods of self-disruption of all communication, sleeplessness and exhaustion (later revealed to be consistent with part of a bigger picture under RDS). Patient also expressed her awareness of the negative impact these were having on her familial and client relationships. In order to further examine the mechanisms of potential addiction, GARS evaluation was performed on 11 December 2020, with a score of 11 prompting a new multi-practitioner coordinated push to reduce dependence. With the results displayed in her GARS test, she carries an array of brain reward genetic antecedents which load onto RDS and associative behaviors including ADD. 

## 5. Discussion

The GARS test recommended KB220PAM (a semi-customized KB220 that offers pro-dopamine regulation by providing a nutraceutical interventional precision complex to balance serotonergic, endorphinergic, glutaminergic, and dopaminergic system restoration). Following this sojourn, the patient has experienced continued improvement as evidenced by the following outcomes: (1) With family collaboration, the patient sought further counsel from the “Mount Sinai Addictive Behavior Program”; (2) Personal coaching focus with a foundation of clarity. Patient began to confront issues including denial, shame, blame, regret, remorse, guilt, hopelessness, helplessness and fear; (3) With the clarity and stabilizing process offered by EFT and pro-dopamine nutrition, the patient progressed to a physician-recommended new step, liberating her from medicinal dependence; (4) With cessation of 95% of medications (with the exception of one stabilizing medicinal agent), along with taking the KB220PAM, the patient confronted and overcame previously unassociated and unaddressed alcohol addiction [74]; (5) Patient’s awareness, clarity, accountability and personal drive has been clearer than she has ever remembered; (6) Her reliability and willingness to be accountable has created opportunities for transforming relationships in her personal and work life that commonly disintegrate permanently for individuals suffering from addiction.

Musculoskeletal pain can lead to primary pharmaceutical exposure and secondary pharmaceutical exposure (surgery, anxiety, depression), leading to a snowball of attempts at palliative care which persist until causative factors are addressed [75]. Pharmacological treatments may delay correction and increase allostatic loading and the development of chronic and equally disabling conditions [76]. It appears that a model of integrated neurological, muscular and skeletal care emphasizing awareness through proprioception and resuscitation of survival processes can lead patients to more complete recovery through more complete corrective care coupling subluxation with H-Wave therapy [28,77]. In this case, rapid recovery from spine-related neurological disruption and a mindset of whole-body correction led to more sustainable natural rewards associated with behavior modification and natural biochemical balance [78,79,80,81,82,83,84,85,86,87,88].

The GARS results certainly provide genetic information suggesting a high risk for RDS-type behaviors. In fact, as is the case for the patient in this study, having eleven polymorphic risk alleles across the brain reward cascade (see Figure 1) support genetically induced addictive-like behaviors, including ADD, Alcoholism and other RDS-driven behaviors magnified by chronic pain.

Solving a central problem of the opioid crisis may involve its redefinition around complexity of pain rather than by chronicity alone. Pain management as an early intervention further sets a poor standard of care which misses the opportunity to connect seemingly unrelated health issues which may undermine complete recovery from stress and trauma. Based on the case, one could conclude more awareness of the subluxation-based model of care is needed for early identification of candidates for care. Focusing on spine-related issues can lead to finding a common thread in care that guides the integration needed to address all pertinent issues. Essential functions, specifically balance and breathing can become indicators for broad applications of this model of care, providing guidance to sequencing care and connecting protocols. More studies should be conducted to strengthen the connection between respiration, natural rewards, the methods we employed and how to leverage this information for better organization of integrative care. 

In summary, in this case study and review of the literature, it seems prudent that the treatment of pain and addiction may require a muti-therapeutic protocol, instead of simply administering powerful analgesics with subsequent unwanted sequalae [89,90]. It is estimated that there are 14,500 active clinics and programs in the United States of America providing treatment for all varieties of addictive behaviors, that we refer to as RDS. While we acknowledge that the majority of them provide needed support with the best intentions for the victims of RDS, we propose herein that most of their efforts, especially during aftercare following higher level rehabilitation when risk of relapse is high, are not based on current scientific evidence.

Not many programs provide evidenced-based treatment options throughout the course of recovery. In our approach, we favor protocols that empower the patient by offering control of intentional thoughts and behaviors, retaining personal responsibility over health driven by greater utilization of genetic and neurophysiologic data. We propose that hypodopaminergic traits (genetic) and/or states (epigenetic) are crucial in terms of continued motivation to use/abuse alcohol and/or other substances and can result in relapse. While evidence for FDA-approved medications in the treatment of drug addiction (e.g., nicotine, alcohol, opiates) exists, these medications tend to favor short-term effects by inhibiting dopamine [91]. Instead, we advocate for the use of long-term benefits that induce “dopamine homeostasis”, or in simpler terms “normalcy”. 

Furthermore, we propose that this could be achieved through a variety of holistic modalities including, but not limited to, meditation, exercise, yoga, hyper-oxygenation, dopamine-boosting diets, heavy metal detoxification, and most importantly, brain neurotransmitter balancing with nutraceuticals (e.g., KB220 variants). In terms of pain relief and correction of its cause, we endorse the non-invasive, non-addictive H-Wave^®^ therapy coupled with chiropractic adjustment of subluxation. 

Certainly, we support 12-step programs and fellowships, but not as a stand-alone treatment option, particularly during aftercare [92]. Additionally, we present some scientific evidence for why resting-state functional connectivity (rsfMRI) is so crucial and could be the key to treating RDS [54]. Given the established role of the resting-state network in internal modes of cognition through utilization of acquired knowledge, with more research we can further define its adaptive value as a homeostatic set point and basis for applying intelligence to healthcare [93]. Since food, smoking, drugs, gambling, and even compulsive sexual behavior can reduce rsfMRI, we postulate that modalities (following required research), that can repair impaired cross talk between various brain regions (e.g., nucleus accumbens, hippocampus, cingulate gyrus, etc.) should be integrated into every aftercare plan. Anything less will inevitability result in the so called “revolving door” syndrome for as many as 90% of patients [20,50,94].

## 6. Conclusions

The take home message is that the utilization of this multi-faceted approach led to functional restoration of previous physical disabilities and cessation of powerful pharmacological agents to treat ADD, anxiety, depression and pain in a female patient experiencing multiple RDS behaviors [27,30,40,61,62,63,93,95,96,97,98,99,100,101]. Due to limited number of subjects, we believe that more studies utilizing this protocol may assist in our understanding of novel ways to provide effective tools to overcome pain and addiction. 

**Figure 3:**
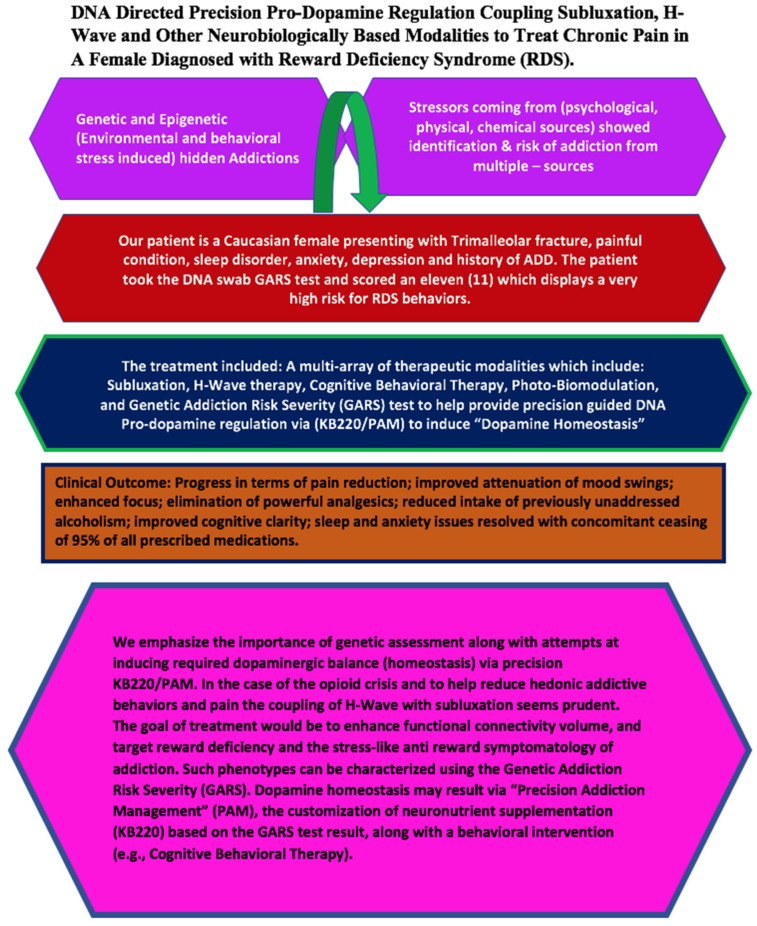
Coupling DNA, Subluxation Repair, H-Wave and Pro-Dopamine regulation in a diagnosed Reward Deficiency Syndrome female patient.

To help us understand this complex protocol, Figure 3 presents a schematic model of these results. 

## Data Availability

Not applicable.
